# GPS/GLONASS Combined Precise Point Positioning with Receiver Clock Modeling

**DOI:** 10.3390/s150715478

**Published:** 2015-06-30

**Authors:** Fuhong Wang, Xinghan Chen, Fei Guo

**Affiliations:** School of Geodesy and Geomatics, Wuhan University, 129 Luoyu Road, Wuhan 430079, China; E-Mail: fhwang@sgg.whu.edu.cn

**Keywords:** GPS/GLONASS, precise point positioning, receiver clock modeling, convergence time, inter-system bias

## Abstract

Research has demonstrated that receiver clock modeling can reduce the correlation coefficients among the parameters of receiver clock bias, station height and zenith tropospheric delay. This paper introduces the receiver clock modeling to GPS/GLONASS combined precise point positioning (PPP), aiming to better separate the receiver clock bias and station coordinates and therefore improve positioning accuracy. Firstly, the basic mathematic models including the GPS/GLONASS observation equations, stochastic model, and receiver clock model are briefly introduced. Then datasets from several IGS stations equipped with high-stability atomic clocks are used for kinematic PPP tests. To investigate the performance of PPP, including the positioning accuracy and convergence time, a week of (1–7 January 2014) GPS/GLONASS data retrieved from these IGS stations are processed with different schemes. The results indicate that the positioning accuracy as well as convergence time can benefit from the receiver clock modeling. This is particularly pronounced for the vertical component. Statistic RMSs show that the average improvement of three-dimensional positioning accuracy reaches up to 30%–40%. Sometimes, it even reaches over 60% for specific stations. Compared to the GPS-only PPP, solutions of the GPS/GLONASS combined PPP are much better no matter if the receiver clock offsets are modeled or not, indicating that the positioning accuracy and reliability are significantly improved with the additional GLONASS satellites in the case of insufficient number of GPS satellites or poor geometry conditions. In addition to the receiver clock modeling, the impacts of different inter-system timing bias (ISB) models are investigated. For the case of a sufficient number of satellites with fairly good geometry, the PPP performances are not seriously affected by the ISB model due to the low correlation between the ISB and the other parameters. However, the refinement of ISB model weakens the correlation between coordinates and ISB estimates and finally enhance the PPP performance in the case of poor observation conditions.

## 1. Introduction

GNSS has been demonstrated to be powerful in positioning, navigation, and timing (PNT) applications in the past decades. Due also to the presence of satellite and receiver clock offsets, GNSS observations are always biased. Processing of double difference observations between pairs of satellites and receivers that are free of clock offsets is thus a popular way in GNSS data processing. However, this approach only allows for the determination of baseline vectors rather than absolute positions. Alternatively, the clock offsets can be estimated as unknowns for single-receiver point positioning. As an emerging positioning technique, precise point positioning (PPP) has been widely applied in geodesy, geodynamic, and remote sensing in the past few years [1,2,3,4,5,6,7,8]. Conventionally, precise clock products provided by the International GNSS Service (IGS) or some other analysis centers (ACs) are employed to eliminate the satellite clock biases. As to the receiver clock biases, they have to be determined at the user ends. Usually, this is done by introducing an additional parameter for every observation epoch [9]. That way even low quality clocks such as small and inexpensive quartz crystal oscillators can be used at the receiver sides [10]. Certainly, we cannot take full advantage of a stable clock when using such a simple method to account for receiver clock errors.

In case the receiver is driven by a stable oscillator, the absolute information as well as the epoch-to-epoch change of the receiver clock may be predictable. Consequently, the temporal variation of the receiver clock bias may be modeled. Although the idea of receiver clock modeling is not really new, it is of little practical use due to the limited stability of internal oscillators at the user ends. Lichten and Border (1987) [11] used polynomial representations for receiver clocks running off hydrogen masters which proved advantageous in orbit and baseline determination when geometry or data were limited. With the increasing number of receivers equipped with high-precision oscillators, a growing interest in receiver clock modeling can be noted in literature of recent years [12,13,14]. Weinbach and Schőn (2011, 2013) [10,15] investigated the feasibility and impact of advanced receiver clock modeling in precise GPS data analysis. The receiver clock on board of the Low Earth Orbiters (LEOs) such as GRACE was modeled by a sequence of piecewise linear parameters and the simulation results indicated that clock modeling reduces the RMS by almost 40% in the radial component. In addition to the modeling of the deterministic behavior, Wang and Rothacher (2013) [16] investigated the stochastic model of high-stability ground clocks and the benefit in kinematic positioning. For excellent clocks, an improvement of a factor of three can be obtained for the kinematic height estimates. Moreover, the use of relative clock constraints allows for a higher time resolution of the zenith tropospheric path delay estimates, which is essential for the determination of water vapor. However, almost all the previous research concerning the receiver clock modeling is mainly limited to the use of single GPS measurements. The fusion of multi-GNSS will undoubtedly increase the number of visible satellites, optimize the spatial geometry and therefore it is expected to further enhance the PPP performance, including the positioning accuracy, reliability, and convergence time [17,18,19,20,21,22]. But the models of multi-GNSS combined PPP with receiver clock modeling as well as the benefits are not exploited yet. Thus more researches are highly desirable. 

In this paper, additional GLONASS observations are applied to augment GPS PPP with receiver clock modeling. The basic mathematic models including the GPS/GLONASS observation equations (functional model), stochastic model, and receiver clock model are briefly introduced in Section 2. Next, the validations are performed in Section 3. Three different schemes are designed to test the effectiveness of the receiver clock modeling using GPS and GLONASS observations. Furthermore, the impacts of inter-system bias models between GPS and GLONASS are investigated. Finally, the summary and conclusions are presented in Section 4.

## 2. Mathematical Models

Different from the traditional PPP model, in which the receiver clock offset parameters are commonly modeled as white-noise processes, herein the GPS/GLONASS combined PPP model with receiver clock modeling is derived for the users equipped with high performance oscillators. The mathematical models including both functional and stochastic models are given in this section.

### 2.1. Functional Model

Taking the time scales difference and receiver clock modeling into account in the observation equations, the ionosphere-free (IF) linear combinations of the GPS/GLONASS combined PPP can be expressed as:

(1) $$P_{IF}^{G}=\rho^{G}+c(\delta t_{0}+dt\cdot \delta t^{\prime })-c\delta t^{G}+m_{trop}^{G}\cdot \delta_{zwd}+\varepsilon_{P}^{G}$$

(2) $$L_{IF}^{G}=\rho^{G}+c(\delta t_{0}+dt\cdot \delta t^{\prime })-c\delta t^{G}+m_{trop}^{G}\cdot \delta_{zwd}+b^{G}+\varepsilon_{L}^{G}$$

(3) $$P_{IF}^{R}=\rho^{R}+c(\delta t_{0}+dt\cdot \delta t^{\prime }+\delta t_{sys})-c\delta t^{R}+m_{trop}^{R}\cdot \delta_{zwd}+\varepsilon_{P}^{R}$$

(4) $$L_{IF}^{R}=\rho^{R}+c(\delta t_{0}+dt\cdot \delta t^{\prime }+\delta t_{sys})-c\delta t^{R}+m_{trop}^{R}\cdot \delta_{zwd}+b^{R}+\varepsilon_{L}^{R}$$

where $P_{IF}^{G},\;L_{IF}^{G},\;P_{IF}^{R},\;L_{IF}^{R}$ represent the IF pseudorange and IF carrier phase observations for GPS and GLONASS denoted by superscript *G* and $1\leq n\leq \text{int}\left(\frac{N-1}{2}\right)$, respectively. It is worth noting that the pseudorange observations should first be corrected by differential code biases (DCBs). *c* is the speed of light in vacuum and ρ represents the geometric range for a specific satellite-receiver link. Assuming the oscillator is stable in a short period, the receiver clock bias can be modeled as a first-order linear function, including one receiver clock time offset δ*t*_0_ and one frequency offset $\delta t^{\prime }$ over the time interval *dt*. The time interval *dt* depends on the frequency stability, which can be quantified by Allan variance. δ*t_sys_* is the time scales difference (or namely inter-system bias, ISB) between GPS and GLONASS. δ*t* denotes satellite clock offset which can be corrected by precise clock products. δ_*zwd*_ is the wet component of zenith tropospheric path delay (ZPD), which is commonly estimated as unknown, while the hydrostatic ZPD can be first corrected by empirical models, such as Saastamoinen model. *m_trop_* is the ZPD mapping function. *b* is the corresponding ambiguity (in length). ε represents the residual errors such as multipath effect and measurement noises. The corrections related to phase wind up, ocean loading, phase center offset and phase center variation, solid earth tide can be conventionally corrected according to Kouba and Héroux (2001) [23].

For a given epoch, the estimates vector *X* includes three positional parameters, two receiver clock offset coefficients, one time scales difference, one zenith tropospheric wet delay, and a set of carrier phase ambiguities. That is:

$$X={\left\{\overset{position}{\overset{︷}{x,y,z}},\underset{receiver\_clock}{\underset{︸}{\delta t_{0},\delta t^{\prime },\delta t_{sys}}},\delta_{zwd},\overset{GPS\_ambiguities}{\overset{︷}{b_{1}^{G},\ldots ,b_{m}^{G},}}\underset{GLONASS\_ambiguities}{\underset{︸}{b_{1}^{R},\ldots ,b_{n}^{R}}}\right\}}^{T}$$

where *m* is the number of visible GPS satellites and *n* is the number of visible GLONASS satellites. For kinematic PPP, the coordinate parameters are generally modeled as white noise process, while the tropospheric delay is modeled as random walk process. The carrier phase ambiguities are assumed to be constants in a continuous arc. The time scale difference can be modeled as white noise process or random walk process or even constant. The influence of different ISB models will be discussed in Section 3.3.

### 2.2. Stochastic Model

Assuming there is no correlation between the code and carrier phase measurements [24], the stochastic model of GPS/GLONASS pseudorange and carrier phase observations can be described as the following equation:

(5) $$Cov(i,j)=\left\{ \begin{array}{l} \begin{array}{cc} \tau^{2}\sigma_{0}^{2} & \left(i=j\right) \end{array}\\ \begin{array}{cc} 0 & \left(i\neq j\right) \end{array} \end{array}\right.\begin{array}{cc} , & \sigma_{0}^{2}=a^{2}+b^{2}{\text{cos}}^{2}E \end{array}$$

where σ_0_ is the standard deviation of raw measurements (unit: m); τ is the noise amplification factor related to the combination coefficients, where τ equals approximately 3 for the IF combinations; *E* is the satellite elevation angle (unit: rad); *a* and *b* are empirical constants. For GPS, *a* and *b* are generally set to be 0.003 mm for carrier phase and 0.3 m for code observations. For GLONASS, the coefficients are increased by a factor of 1.5 due to the less accurate satellite orbit and clock products compared to GPS.

### 2.3. Receiver Clock Model

The Allan variance or Allan deviation is a measure of frequency stability in clocks, oscillators and amplifiers. They are often used to visualize graphically the random characteristics of clock behavior [25]. In this contribution, a modified Allan variance is utilized to identify the dominant noise type from the slope plotted on a double-logarithmic scale, especially for the white phase noise and flicker phase noise. The modified Allan variance $\sigma_{A}^{2}(\tau )$ could be estimated in terms of δ*t*(*i*) by

(6) $$\text{Mod}\;{\hat{\sigma }}_{A}^{2}(n\cdot \tau_{0})=\frac{1}{2n^{4}\tau_{0}^{2}(N-3n+1)}\cdot \sum_{j=1}^{N-3n+1}{\left(\sum_{i=j}^{j+n-1}\left(\delta t_{i+2n}-2\delta t_{i+n}+\delta t_{i}\right)\right)}^{2}$$

where δ*t_i_* is clock bias at epoch *i*, *N* the number of samples, τ_0_ the sampling interval, and *n* the smoothing factor, which can be taken as $1\leq n\leq \text{int}\left(\frac{N-1}{2}\right)$.

Apart from the characterization of the frequency stability, it can also be used to estimate the accumulated time error of a clock, *i.e.*, the error of the predicted clock reading at some time due to random frequency errors. With the time prediction error, we can determine the proper modeling interval. In view of the potential short-term correlation among receiver clock biases, a two-dimensional state model is employed to depict the dynamic process of receiver clock offset, and the state transition equations can be expressed as:

(7) $${\left[\begin{array}{l} \delta t_{0}\\ \delta t^{\prime } \end{array}\right]}_{\text{k}}=\left[\begin{array}{cc} 1 & dt\\ 0 & 1 \end{array}\right]{\left[\begin{array}{l} \delta t_{0}\\ \delta t^{\prime } \end{array}\right]}_{k-1}+\left[\begin{array}{l} \omega_{0}\\ \omega^{\prime } \end{array}\right]$$

(8) $$Q_{\bar{x},k}=\Phi_{k,k-1}Q_{\hat{x},k-1}\Phi_{k,k-1}^{T}+Q_{\omega ,k}$$

where $\omega_{0},\omega^{\prime }$ represent the corresponding process noises, $Q_{\hat{x}}$ is the covariance matrix, Φ_*k*,*k*−1_ is the transition matrix. According to Brown and Hwang (2005) [26], *Q*_ω,*k*_ can be expressed as:

(9) $$Q_{w,k}=\left[\begin{array}{cc} \frac{h_{0}}{2}dt+2h_{-1}{\left(dt\right)}^{2}+\frac{2}{3}\pi^{2}h_{-2}{\left(dt\right)}^{3} & \frac{h_{0}}{2}+2h_{-1}dt+\frac{2}{3}\pi^{2}h_{-2}{\left(dt\right)}^{2}\\ \frac{h_{0}}{2}+2h_{-1}dt+\frac{2}{3}\pi^{2}h_{-2}{\left(dt\right)}^{2} & \frac{h_{0}}{2dt}+4h_{-1}+\frac{8}{3}\pi^{2}h_{-2}dt \end{array}\right]$$

where *h*_0_, *h*_−1_, *h*_−2_ refer to the spectral power density of white frequency noise, flicker frequency noise, random walk frequency noise, respectively. Compared to a common method where the flicker noise contribution is neglected [27], the matrix includes an approximation of the impact of flicker frequency noise, which cannot be modeled exactly by a finite-order state model [28].

## 3. Performance Evaluations

In order to evaluate the performance of GPS/GLONASS PPP with receiver clock modeling, a week of (1–7 January 2014) multi-GNSS data at an interval of 30 s were first collected from several IGS stations equipped with high-precision atomic clocks, and then processed with the following three models:

Model 1: GPS-only PPP with receiver clock modeling;
Model 2: GPS/GLONASS PPP without receiver clock modeling;
Model 3: GPS/GLONASS PPP with receiver clock modeling.

The precise satellite orbit and clock products from ESA were used. The elevation mask angle was set to 7°. The positioning results were compared with the IGS published coordinates [29]. In general, the reference coordinates have an accuracy of few millimeters.

### 3.1. Accuracy and Reliability Analysis 

**Figure 1:**
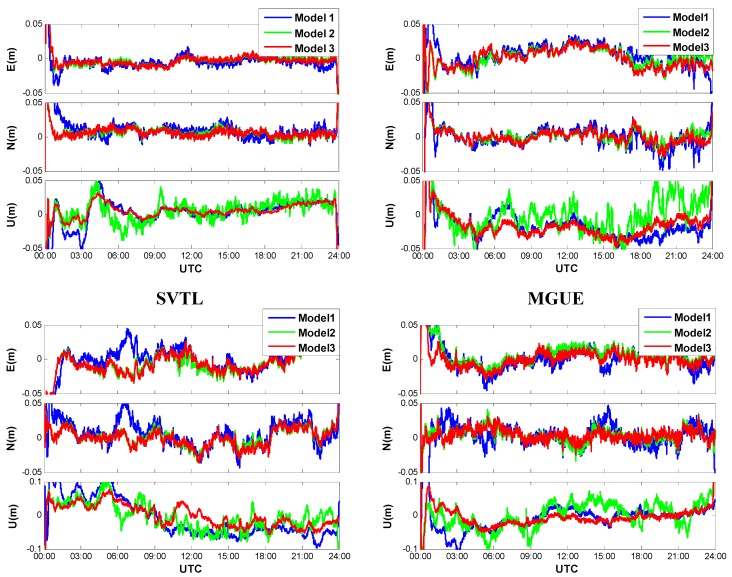
Positioning errors of kinematic PPP with forward Kalman filter.

Figure 1 shows that the kinematic positioning results of four IGS tracking stations (namely SVTL, MGUE, HOB2 and WTZR) with the forward Kalman filter (KF). One may notice that Model 1 shows the worst performance, whereas Model 3 shows the best performance. Compared to the GPS-only PPP (Model 1), solutions of the GPS/GLONASS combined PPP (Models 2 and 3) are much better no matter if the receiver clock offsets are modeled or not, indicating that the positioning accuracy and reliability are significantly improved with the additional GLONASS satellites. The improvement can be pronounced particularly in the case of insufficient number of GPS satellites or poor geometry conditions. Solutions of the Model 3 run more stable and smoother than those of the Model 2, especially for the height components. In other words, the positioning accuracy and reliability are improved once the receiver clock modeling algorithm is employed. This is reasonable when we acknowledge the fact that the correlation between the horizontal coordinate and the receiver clock offset is generally small, while the coordinate of the height component is highly correlated with the receiver clock offset. With receiver clock modeling, the process noise can be significantly reduced thus a higher degree of temporal decorrelation of the different parameters can be achieved. 

**Figure 2:**
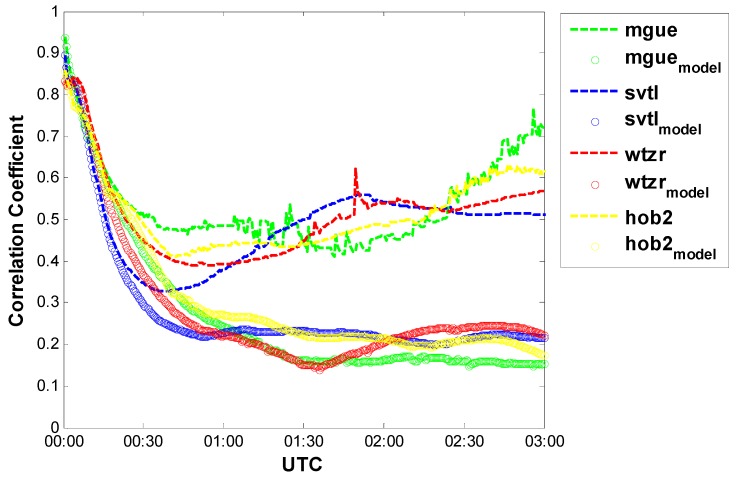
Correlation coefficients between the receiver clock offset and height coordinate (Model 2 *vs.* Model 3).

To confirm this, Figure 2 shows the correlation coefficients between the receiver clock offset and height coordinate, which can be calculated by the following equation.

(10) $$\rho =\frac{\text{cov}(\delta u,\delta t_{0})}{\sqrt{\sigma_{\delta u}^{2}\cdot \sigma_{\delta t_{0}}^{2}}}$$

where ρ represents the correlation coefficient, cov(δ*u*, δ*t*_0_) denotes the covariance between receiver clock offset and height coordinate, $\sigma_{\delta u}^{2},\sigma_{\delta t_{0}}^{2}$ represent the variances of the height coordinate and receiver clock offset, respectively. Obviously, for Model 3, the correlation decreases gradually over time and finally converges to a much smaller value compared to Model 2.

**Figure 3:**
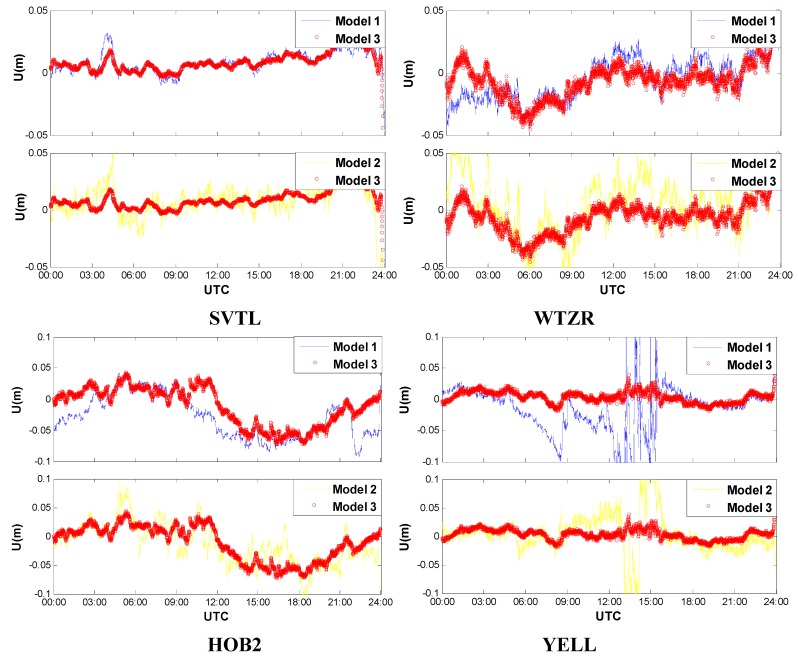
Vertical positioning errors of kinematic PPP with forward and backward smoothing Kalman filter.

Comparisons of the smoothed (forward and backward smoothing KF) station height are presented in Figure 3 to further verify the superiority of multi-system combination and receiver clock modeling. Similarly, the GPS/GLONASS combined PPP outperforms the GPS-only PPP due to the increased number of visible satellites and improved geometry. This is particularly pronounced for the YELL station during the period of Universal Time Coordinated (UTC) 6–18 (from UTC 06:00 to UTC 08:00). Comparing with the Model 2 and Model 3, also it can be concluded that the vertical positioning accuracy and stability are significantly enhanced with the receiver clock modeling.

**Figure 4:**
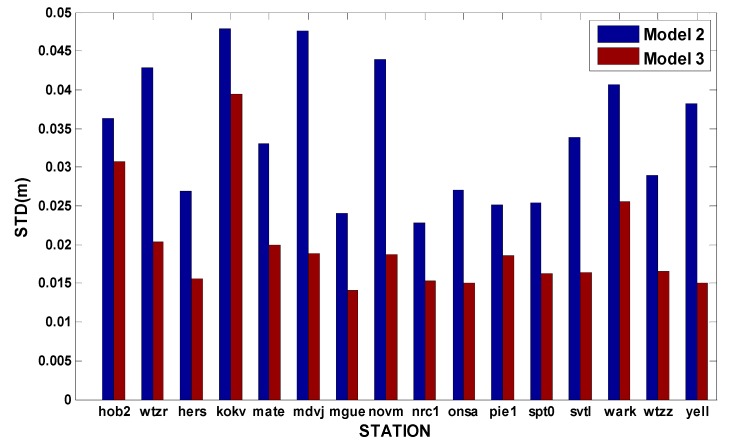

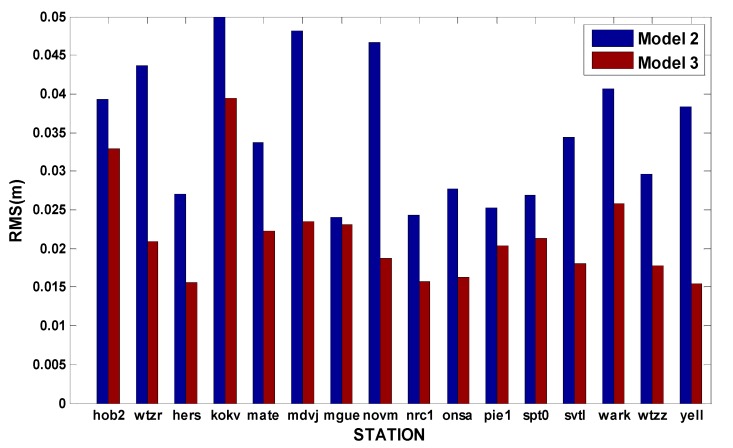
The STDs and RMSs of the kinematic GPS/GLONASS PPP (Model 2 *vs.* Model 3).

Moreover, the statistic standard deviations (STDs) and root mean squares (RMSs) are given in Figure 4 based on the test of Model 2 and Model 3 with a variety of IGS stations on 1–7 January 2014. As shown in Figure 4, the STDs and RMSs of the Model 3 are much smaller than those of Model 2. The average RMS improvement reaches approximately 36%. Sometimes, the RMS improvement can be around 60% for specific stations. It is demonstrated that receiver clock modeling can exploit the additional information concerning the frequency stability of high-precision oscillators and finally improve the positioning accuracy.

### 3.2. Convergence Analysis

**Figure 5:**
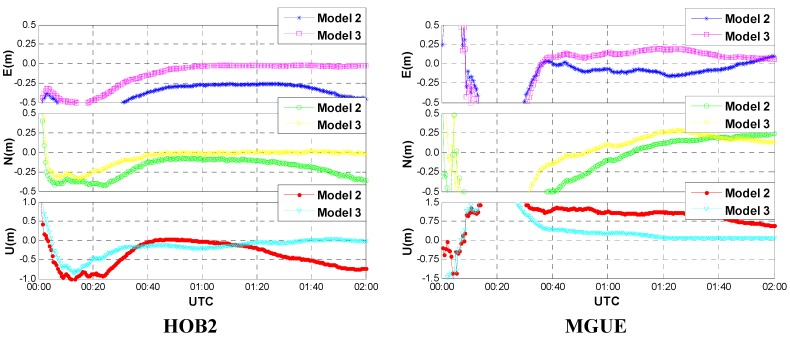

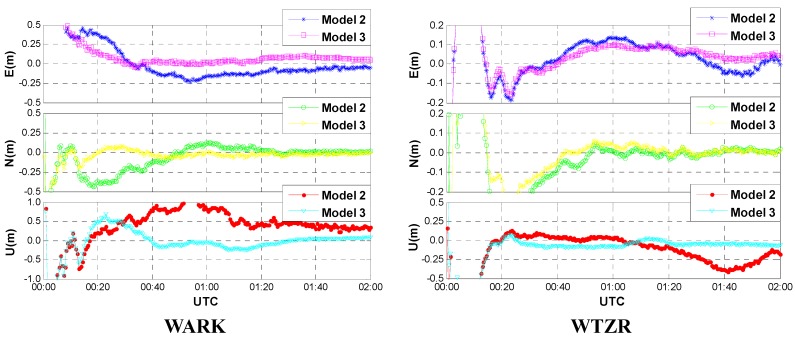
Convergence performances of the kinematic GPS/GLONASS PPP (Model 2 *vs.* Model 3).

To investigate the benefits of receiver clock modeling for PPP convergence, the same data were reprocessed with the first two hours’ (UTC 0–2) data and reserved only a few valid satellites (approximately three GPS and three GLONASS) to simulate the severe observation environment, which is common in real kinematic sceneries. Figure 5 shows the forward kinematic positioning errors of GPS/GLONASS combined PPP with Model 2 and Model 3. Obviously, the positioning accuracy of the Model 3 is better than that of the Model 2 at the initial stage, thus leading to a shorter convergence time. The positioning accuracy can even be improved by around 0.5 m and 1.0 m in the horizontal and vertical, respectively, for a certain period of time. Moreover, the results of Model 3 are more stable than those of Model 2 after convergence, which indicates that the receiver clock modeling can partly overcome the problem of filter divergence due to the poor geometry. Therefore, the PPP with receiver clock modeling is more capable of enhancing the resistance to terrible observation environments than the conventional PPP.

**Figure 6:**
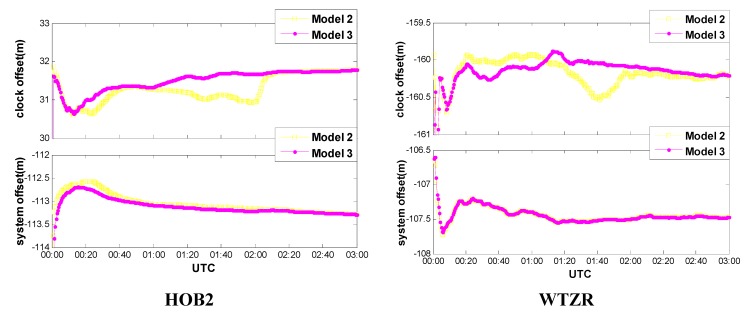
Receiver clock offset and ISB of GPS/GLONASS combined PPP.

Figure 6 shows the estimated receiver clock offsets and the GPS-GLONASS time scale differences for the first 3 h on HOB2 and WTZR stations. As shown in Figure 6, the estimated receiver clock biases of both Models do not change frequently, which further confirms the feasibility of receiver clock modeling. Since the receiver clock bias parameters are estimated with different schemes, the estimates consequently perform different characteristics. Take the Model 2 for example, the receiver clock estimates run with larger variations since the potential short-term correlations among the receiver clock biases are ignored. However, the receiver clock estimates run much smoother if they are modeled as a linear model in a specific segment. One may notice that, the receiver clock estimates in Figure 6 show similar features as the vertical coordinates in Figure 5. This can be attributed to the correlation between the receiver clock bias and station height as mentioned in Section 3.1. As to the time scale differences, estimates from both models are close to each other because the same appropriate model is utilized in Figure 6.

### 3.3. Impacts of Different Inter-System Bias Models

Figure 6 shows that the estimates of time scale difference are stable during a period of several hours, suggesting that in addition to the receiver clock bias modeling, modeling refinements on the inter-system bias (ISB) are expected to further improve the performance of GPS/GLONASS combined PPP. Therefore, the following three different schemes were designed to test the impacts of inter-system bias modeling on PPP solution [30,31].

Scheme 1: GPS/GLONASS PPP, the ISB is modeled as constant daily;
Scheme 2: GPS/GLONASS PPP, the ISB is modeled as white noise process;
Scheme 3: GPS/GLONASS PPP, the ISB is modeled as random walk process.

**Figure 7:**
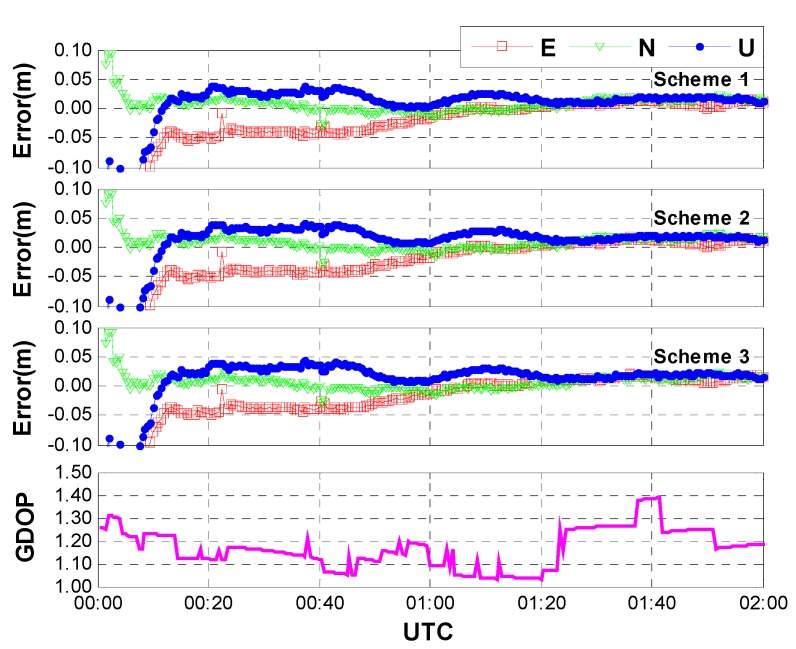
Positioning error and GDOP of GPS/GLONASS combined PPP with different ISB models (case one: sufficient number of GPS and GLONASS satellites, an average of 15 satellites included).

**Figure 8:**
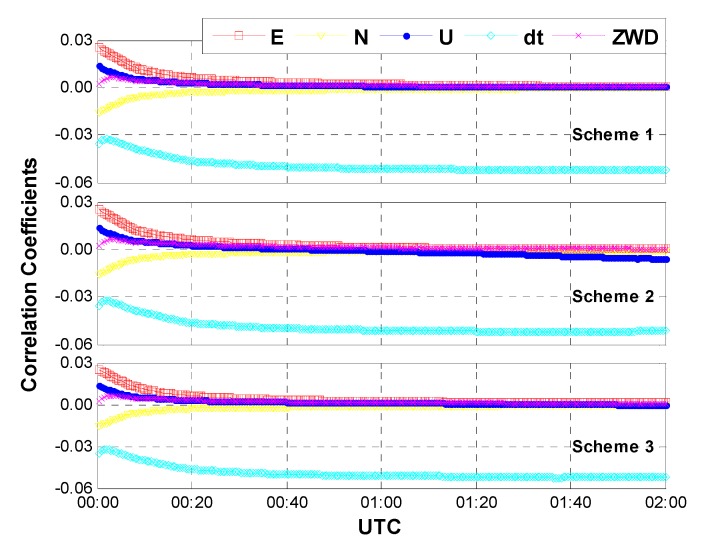
Correlation coefficients between ISB and other parameters (case one: sufficient number of GPS and GLONASS satellites, an average of 15 satellites included).

**Case one:** The same data used in Section 3.1 were processed with the above three schemes. In this case, an average of 15 GPS/GLONASS satellites are available. Figure 7 shows the positioning results and the corresponding geometric dilution of precision (GDOP) for the first 2 h on HOB2. The other stations show the similar results and thus are not presented herein. After a short time convergence, both the horizontal and vertical components reach an accuracy of a few centimeters (±5 cm). One may notice that the performances of the three different schemes are almost the same. In other words, the positioning accuracy is not seriously affected by the different models of inter-system bias. This is reasonable when we acknowledge the fact that the correlation coefficients between IBS and other parameters are quite small due to the sufficient number of satellites and the pretty good geometry. As shown in Figure 8, the correlation coefficients between ISB and receiver clock bias show the largest values (about 0.05), whereas the correlation coefficients between ISB and the other parameters (such as coordinates and tropospheric delay) are close to zeroes. Moreover, the correlation coefficients seem to be unaffected by the adjustment of inter-system bias models. 

**Figure 9:**
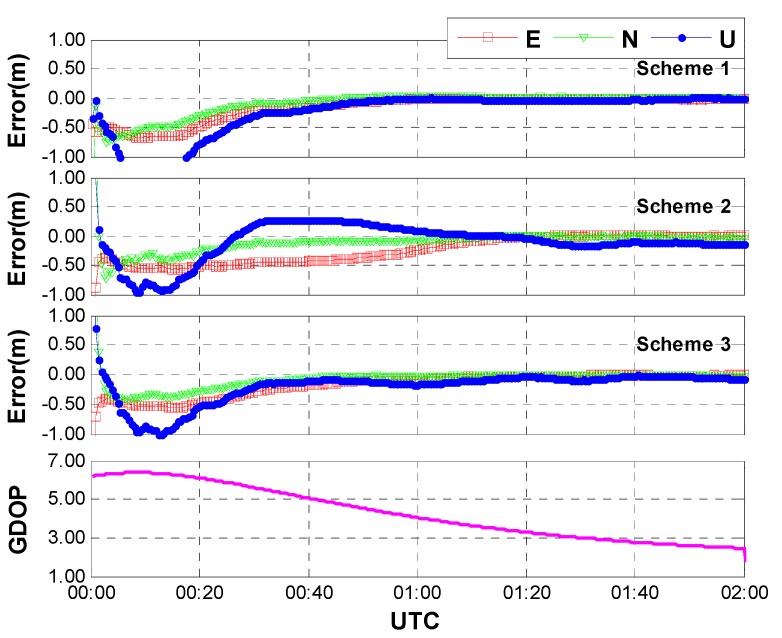
Positioning error and GDOP of GPS/GLONASS combined PPP with different ISB models (case two: limited number of GPS and GLONASS satellites, an average of 6 satellites included).

**Figure 10:**
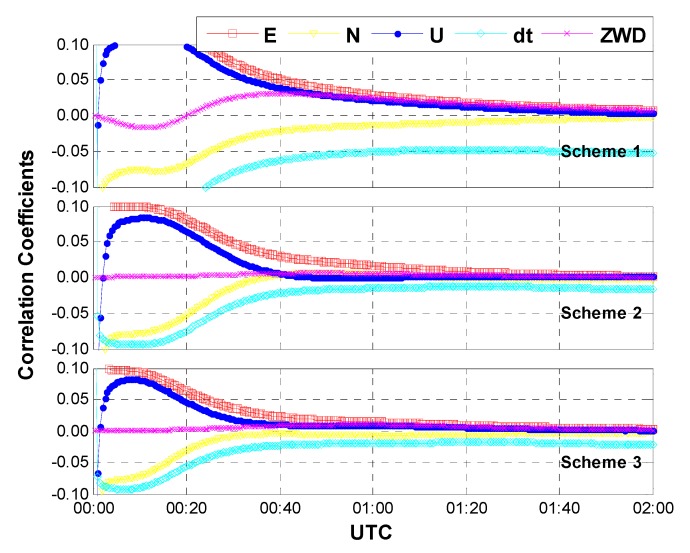
Correlation coefficients between ISB and other parameters (case two: limited number of GPS and GLONASS satellites, an average of 6 satellites included).

**Case two:** Likewise, the same data used in Section 3.2 were processed with the above three schemes. As mentioned in Section 3.2, only a few satellites (3G + 3R) are available in this case. Figure 9 shows the positioning results and the corresponding GDOP for the first two hours on HOB2, and the correlation coefficients between the ISB and other parameters are shown in Figure 10. Due to the limited number of satellites and poor geometry, the positioning accuracy decreases to a few decimeters. Comparing with the positioning errors of the three schemes, we can find that the latter two schemes outperform the first one, indicating the GPS/GLONASS combined PPP can benefit from the inter-system bias modeling. This is true at the initial stage, especially the improvement of convergence. The correlation coefficients in Figure 10 show that the coordinates as well as receiver clock biases are highly correlated with the ISB estimates in the case of an insufficient number of satellites and poor geometry. Once the ISBs are modeled as random walk noise or white noise, the correlation coefficients can be obviously reduced. That means the parameters can be better separated from each other, and consequently we can obtain more accurate and reliable solutions.

## 4. Conclusions

Receiver clock modeling has been introduced into GPS/GLONASS combined PPP in this contribution. Firstly, the basic mathematic models including the observation model, stochastic model, and receiver clock model are presented. Thereafter, three different schemes are designed to test the performance of GPS/GLONASS combined PPP with receiver clock modeling. Results show that the positioning accuracy of the GPS/GLONASS combined PPP are better than that of GPS-only PPP, particularly in the case of insufficient number of GPS satellites or poor geometry conditions. Compared with the traditional PPP, the new PPP with receiver clock modeling is capable of improving the positioning accuracy. This is particularly pronounced for the vertical coordinates. With receiver clock modeling, the highly correlated parameters between the station height and receiver clock offset decrease significantly. Statistic RMSs show that the average improvement of three-dimensional positioning accuracy reaches 30%–40%. In addition, the convergence time can be reduced by introducing receiver clock modeling into GPS/GLONASS PPP in severe environments.

In addition to the receiver clock modeling, the influence of inter-system bias (ISB) models are investigated. Results show that the positioning accuracy is not seriously affected by the different models of inter-system bias in the case of sufficient number of satellites with good geometry. This is reasonable when we acknowledge the fact that the correlation coefficients between IBS and other parameters are quite small in this case. However, the coordinates as well as receiver clock biases are highly correlated with the ISB estimates in the case of insufficient number of satellites and poor geometry. Refinement of the ISB model can better separate the estimates from each other, and consequently enhance the GPS/GLONASS combined PPP.
